# Interstate Reciprocity in Licensing1Answer to prize question No. XLV. New York Medical Journal series of prize essays for which the prize of $25 was awarded. Reprinted from New York Medical Journal Jannary 20, 1906.

**Published:** 1906-07

**Authors:** William Warren Potter

**Affiliations:** Buffalo, N. Y.


### Interstate Reciprocity in Licensing.[1]

BY WILLIAM WARREN POTTER, M. D. BUFFALO, N. Y.

HARDLY a dozen commonwealths had established requirements for State license to practise medicine when the propriety of interstate endorsement without further examination began to be agitated. One of the early suggestions was a national examining board, but this was abandoned very soon by the most careful observers, because it was apparent that the general government would not usurp the police power of the states for the purpose named.

The difficulties which lie across the path of reciprocity arise from the very nature of our political division into States and Territories, united by constitutional provision into a compact whole for certain specified purposes, each division, however, retaining its autonomy for the essential conduct of its internal affairs and for its daily needs.

The general government retains the sole right to declare war, make peace, establish tariff, coin money, maintain post offices and post routes, and to deal with other national necessities. The states retain the right to control their internal affairs, establish police regulations, maintain highways, and to do all things necessary to keep our citizens in a condition of peace and prosperity.

The Washington government will not undertake to administer upon questions that are so purely within the rights and powers of the states as are all matters relating to medical practice; nor will the states yield to the national authorities the control of such purely local subjects as granting license to practise medicine.

The medical practice laws in a greater number of the states contain provisions for the recognition or endorsement of licenses issued by other commonwealths which have standards not lower than the state granting the license. This parity is not always easily established, for the reason that some states maintain a high standard regarding one or two requirements, while exacting a comparatively low test in others, the average being less than a properly graded minimum criterion.

---

1. Answer to prize question No. XLV. New York Medical Journal series of prize essays for which the prize of $25 was awarded. Reprinted from New York Medical Journal Jannary 20, 1906,

Again, all the state medical laws contain provisions prohibiting discrimination against their own citizens in this respect; hence it is impossible for a licensee from one of the lower standard states to obtain indorsement from a state maintaining a higher grade of requirements. Such an one, under existing conditions, must needs take a new examination if he desires to practise in a state of the latter class. How can a reexamination be avoided by a physician with a license of one state who wishes to change his residence and practice to another state? Or, in the language of the question at the head of this brief discourse propounded by the editor of this journal, how may interstate reciprocity be best accomplished?

Having used about half the space allotted for this disquisition in stating the chief obstacles to reciprocity, I will endeavor in the remaining half to answer the question which seems to be uppermost in the minds of many state licentiates. One way to establish universal reciprocity would be to persuade the state legislatures to amend their statutes to a substantial uniformity of diction and requirement. But, as conditions vary in nearly all the political divisions, this may be difficult of accomplishment. Moreover, the process of unifying the statutes, even admitting its possibility, would prove slow and wearisome. It would entail an enormous outlay of time and money, not to mention that patience and endurance would be taxed even to exhaustion.

Fortunately, however, there remains a sure road to reciprocity through the dignified action of a resourceful and selfsacrificing profession. I refer to the unification of standards for admission to study, for teaching the neophyte, and for applying the final state test. In other phrase, when the preliminary or entrance requirements, the length of terms and methods of collegiate training, and the licensing examinations are placed upon a uniform basis throughout the country, the problem of reciprocity in medical licensure will become solved. This, moreover, would appear to be the only equitable solution of the perplexing question.

Of what avail is reciprocity unless founded on justice? Surely a forced interchange of licensing courtesies would be worse than none; it would be irritating, unfair and ephemeral; whereas, if founded upon uniformity of standards, it would be pleasing, just and enduring.

It seems strange, almost absurd, that entrance requirements have not been placed already upon a basis of uniformity. It is wellnigh scandalous that it has not been established. Ignorance should not be tolerated anywhere among those who contemplate entering upon the study of medicine, and the colleges throughout the length and breadth of the land should agree without unnecessary delay upon the minimum of preliminary or entrance qualifications. This once done, it would be comparatively a short step to a standardizing of the methods of teaching, as well as the length of the undergraduate terms. Finally, with these two essentials agreed upon, the licensing boards would be compelled to establish a uniformity of methods of examinations, and, likewise, the states could no longer withold indorsement of licenses.

The Association of American Medical Colleges can be depended upon to establish the first two propositions—namely, the uniformity of entrance requirements and the length of collegiate terms and methods of teaching. It will then be "up to" the examining boards and the state authorities, to use a trite expression of the day, to see that their methods become of uniform standard and that reciprocity is established with promptitude. These later questions can and, without doubt, will be dealt with satisfactorily and conclusively by the National Confederation of State Medical Examining and Licensing Boards, just as soon as the college association settles the two propositions that are before it for consideration. The conference in Chicago last April made substantial progress in this direction, and at its next meeting will most likely reach conclusive action on the subject.

I am persuaded, from considerable observation and experience, that interstate reciprocity in licensing is best accomplished through the avenues named, and, indeed, that only in this way can it be accomplished with fairness to all the variant interests, with lasting credit to the profession, and with substantial benefit to the licensees who expect to derive advantages from this privilege.

284 FRANKLIN STREET.

